# Correlation analysis between sleep quality and the mental health status of female sex workers during the COVID-19 pandemic in Hubei Province

**DOI:** 10.3389/fendo.2023.1193266

**Published:** 2023-07-17

**Authors:** Piyong Zhai, Hao Liu, Yutong Zhang, Tao Huang, Change Xiong, Yang Liu, Guiping Wang, Xin Chen, Jianhua Tan, Chengjun Jiao, Jianbo Zhan, Jing Cheng

**Affiliations:** ^1^ School of Public Health, Wuhan University of Science and Technology, Wuhan, Hubei, China; ^2^ Institute of Health Inspection and Testing, Hubei Provincial Center for Disease Control and Prevention, Wuhan, Hubei, China; ^3^ Division of infectious disease, Chinese Center for Disease Control and Prevention, Beijing, China; ^4^ Institute for the Prevention and Control of Infectious Diseases, Huanggang Provincial Center for Disease Control and Prevention, Huanggang, China

**Keywords:** female sex workers, sleep quality, mental health status, COVID-19, correlation analysis

## Abstract

**Objective:**

Female sex workers (FSWs) in entertainment venues are subject to condemnation and rejection by their families and the outside world. As a result, they are prone to psychological problems, including anxiety and even suicidal tendencies. The aim of the current study was to understand the sleep and mental health status of local FSWs and to identify associated risk factors, so as to provide a scientific basis for improving the social recognition of FSWs and establishing effective psychological interventions.

**Methods:**

A stratified cluster random sampling method was used to select women engaged in commercial sex in bathing, sauna, karaoke halls and other entertainment venues. A self-designed questionnaire assessing mental health-related factors was administered face-to-face with the respondents. 90 participants were randomly selected for blood tests to analyze the relationship between biological indicators and sleep quality.

**Results:**

The rates of depression, anxiety and somnipathy among FSWs were 32.7%, 43.1% and 8.6%, respectively. The correlation coefficients (r) between sleep quality and depression, anxiety and social support were 0.07, 0.09 and -0.09, respectively. Divorce or widowhood, technical secondary school education, alcohol consumption and exercise were independent risk factors for depression in FSWs (p< 0.05) while living in urban areas and counties and having a sense of social support were protective factors (P< 0.05). Quarantining due to the pandemic and exercise were independent risk factors for anxiety in FSWs (P< 0.05) while living in counties and having a sense of social support were protective factors (P< 0.05). Quarantining due to the pandemic was an independent risk factor for somnipathy in FSWs (P< 0.05) while being married was a protective factor (P< 0.05). NE/NA was a protective factor for sleep disorders (OR=0.042, P=0.05), with an AUC of 0.87.

**Conclusion:**

FSWs should appropriately adjust their work form during the pandemic period, maintain a positive and optimistic attitude, establish long-term stable social relationships, and seek a greater sense of social support. The government should provide comprehensive bio-psycho-social interventions to address the mental and physical health status of this population.

## Highlights

- The correlation coefficients (r) between sleep quality and depression, anxiety and social support were 0.068, 0.089 and -0.086, respectively.
- Divorce or widowhood, technical secondary school education, alcohol consumption and exercise were independent risk factors for depression in FSWs.
- Quarantining due to the epidemic and exercise were independent risk factors for anxiety in FSWs.
- Quarantining due to the epidemic was an independent risk factor for somnipathy in FSWs.
- NE/NA was a protective factor for sleep disorders.

## 1 Introduction

Female sex workers (FSWs) in entertainment venues provide sexual services; these services are also known as illicit prostitution or unlicensed prostitution. Sex workers in hotels, restaurants, saunas, bathing nightclubs, clubs, karaoke halls and dance halls are considered high-grade FSWs, and are mainly viewed as a form of entertainment; sex workers in hair salons/beauty salons, hair washing rooms, leisure houses and foot washing houses are considered mid-grade FSWs, and are mainly viewed as providing life services; sex workers in roadside stores, small hotels, guest houses and standing piles are considered low-grade FSWs, and are mainly viewed as providing sexual services; street mobile FSWs are FSWs that are active in the streets, construction sites, markets and other non-fixed places and provide commercial sexual services. FSWs are the high incidence groups of syphilis, Acquired Immune Deficiency Syndrome (AIDS) and other Sexually Transmitted Diseases (STDs), and are also the bridge for the spread of STDs from key populations to the general population

(1). In the present day, the sex work profession has become a more common social phenomenon, and some countries around the world have legalized the sex trade. For example, in the Netherlands, FSWs have access to preventive health services provided by the local public health service and are protected by law (2). However, in most countries, including China, the sex trade is still not considered legal. Because the behaviour of FSWs is contrary to the law and social mainstream, FSWs are mostly referred to as “unlicensed prostitutes”. As a result, they are subject to condemnation and rejection by their families and the outside world and are prone to psychological problems such as anxiety and even suicidal tendencies (3, 4).

A cross-sectional study in the Islamic region revealed that approximately 62.5% of FSWs have mental health problems, with mood disorders and anxiety disorders being the two most common mental health problems (5). In 2022, a longitudinal study in Kenya showed that while mental health problems among local FSWs had declined, suicidal behaviour remained high; the prevalence of mental health problems was 29.9% and the prevalence of suicidal behaviour was 10.2% prior to the commencement of psychological interventions for FSWs (6). FSWs in Myanmar are more vulnerable to the threat of violence from employers due to the lower status of women in this country, which exacerbates their anxiety and depressive symptoms (7). A recent meta-analysis of FSWs in 28 countries indicated that nearly 27% of FSWs have had suicidal thoughts and 20% of FSWs have attempted suicide (8). In China, the rate of psychological disorders among FSWs ranges from 35% to 60% (9–12). However, despite the high prevalence of severe psychological problems, FSWs rarely seek help for their mental health. A Swiss study demonstrated that sex workers receive less health care than the general population and that a feeling of shame is a major barrier to the use of mental health services by FSWs (13). While conventional care is not an effective psychological intervention for FSWs, improving the self-esteem, self-efficacy and coping skills of this population can effectively improve the mental health status and well-being of FSWs (14). However, in China, FSWs are a unique group of marginalized people. To date, few studies have been conducted on the psychological state of FSWs in China, and importantly, most FSWs work in concealed or hidden workplaces and are reluctant to participate in research; thus, many of the available studies are not comprehensive or objective. Moreover, the illegal nature of prostitution in China and the imbalance between the obligations and rights of the CDC(Centers for Disease Control) impede research on and interventions for FSWs (15). The aim of this study was to investigate the mental health status and sleep quality of FSWs in Huanggang City, Hubei Province, and to identify the related risk factors. The ultimate goal of this research was to provide a scientific basis for improving the social recognition of FSWs and establishing effective psychological interventions.

## 2 Materials and methods

### 2.1 Research subjects

This study was conducted in conjunction with HIV sentinel surveillance, and the subjects for this study were women engaged in the commercial sex trade in a community in Huanggang City. Participants were recruited from June to November 2022 and included street itinerant FSWs as well as fixed low-, medium- and high-grade FSWs. All subjects provided written informed consent before taking part in the study.

### 2.2 Survey method

The survey adopted a cross-sectional research design with stratified cluster sampling method. According to the distribution of entertainment venues in different districts (counties) of Huanggang City, a distribution map was drawn and roughly divided into 13 survey areas. The survey areas were stratified according to low-, medium- and high-grade, and a certain number of survey areas were randomly selected in each layer according to a certain proportion. 8 survey areas were finally included in the study, and 8 investigation teams were respectively responsible for investigating all FSWs that could be accessed in the survey area. This on-site investigation was conducted at each participant’s place of work, with strict adherence to the principles of informed consent, anonymity and confidentiality. After obtaining informed consent from the survey respondents, blood was collected from them in a relatively independent space after a face-to-face questionnaire.

According to previous studies, the rate of psychological disorders among FSWs is 62.5% (5). Thus, sample size estimation for the current study was performed as follows $\;n=\frac{Z^{2}p\left(1-p\right)}{\delta^{2}}$ : is the sample size, Z is the statistic for the chosen significance level, p is the incidence of psychological disorders and δ is the tolerance error), where Z was set at 1.96 (α=0.05) and δ=p×10%=0.0625. Accordingly, the estimated required sample size for the current study was 230. To account for participants lost to follow-up and invalid samples, a sample not less than double this minimum number was required (i.e., 460). In total, 830 questionnaires were distributed and 829 were recovered, with a recovery rate of 99.88%. Three invalid questionnaires were excluded, leaving 826 valid questionnaires. Thus, the effective response rate was 99.52%.

### 2.3 Questionnaire

The questionnaire was distributed to all eligible FSWs at each recruited FSW site. The questionnaire contained questions to assess the participants’ basic demographic characteristics; mental health status; sleep quality; knowledge of hepatitis C, syphilis and AIDS prevention; behavioural and lifestyle habits; and dietary and nutritional health status.

#### 2.3.1 Mental health status

The Self-rating Depression Scale (SDS) and Self-rating Anxiety Scale (SAS) were used to assess depression and anxiety. Cronbach’s alpha of SDS and the intraclass correlation coefficient of SAS were 0.87 (16) and 0.92 (17), respectively. The SDS and SAS are commonly used in psychopharmacological studies. Each scale comprises 20 questions and subjects are required to independently respond to each, with completion of each scale taking approximately 10 minutes. The score for each question is then summed, multiplied by 1.25, and rounded to a whole number to obtain a standard score. Higher scores are associated with more severe symptoms of anxiety or depression. The upper limit of the normal range of SDS standard scores is 53 points;< 53 is considered normal, 53-62 reflects mild depression, 63-72 reflects moderate depression, and > 72 reflects severe depression. The upper limit of the normal range of SAS standard scores is 50 points;<50 is considered normal, 50-60 reflects mild anxiety, 61-70 reflects moderate anxiety, and >70 reflects severe anxiety.

#### 2.3.2 Sleep quality

The Pittsburgh Sleep Quality Index (18) (PSQI) was used to assess participants’ sleep quality. The PSQI was compiled in 1989 by Dr. Buysse, a psychiatrist at the University of Pittsburgh. The sensitivity and specificity of the index for cases and normal subjects are 98.3% and 90.2% (Kappa=0.89, *P*<0.01), respectively. This scale is suitable for evaluating the sleep quality of patients with sleep disorders and/or psychiatric disorders as well as the sleep quality of the general population. The scale assesses a participant’s sleep quality over the last month. The higher the score, the worse the sleep quality; a PSQI score > 7 is considered to reflect the presence of a sleep disorder.

#### 2.3.3 Knowledge of hepatitis C and AIDS prevention

With reference to the instruction in the Manual for the Use of the Chinese AIDS Prevention and Control Supervision and Evaluation Framework (Trial) (19), the questionnaire contained eight questions to assess hepatitis C and AIDS prevention knowledge, respectively. Participants who answered six or more questions correctly were considered to have good knowledge; otherwise, they were considered to have poor knowledge (**Supplementary Tables 1, 2**).

#### 2.3.4 Social support rate scale

Social support is an important concept in social psychology; it refers to an individual’s social connections, social integration and main group relationship. Social support can enhance self-awareness and help alleviate an individual’s psychological barriers. This study evaluated the social support perceptions of the study participants using the Social Support Rating Scale (SSRS), a scale designed to determine how much support an individual receives from family, friends and the social environment. The scale measures subjective support (number of friends who offer help, relationship with neighbours, relationship with colleagues, level of support from family), objective support (living situation in the past year, avenues of resolution in emergencies, source of psychological comfort when experiencing stress or resistance) and support utilization (expression when experiencing difficulties). The total SSRS score is the sum of these three subscale scores, with higher scores indicating a higher level of social support (20).

#### 2.3.5 Disease history and behavioural lifestyle

The questionnaire contained self-designed items to assess each participant’s disease history, including whether the participant suffered from diseases of the digestive system, cardiovascular system, respiratory system, reproductive system and others. Current drug treatments were also assessed.

The frequencies of smoking, alcohol consumption, tea consumption and coffee consumption were also investigated, in addition to the participant’s living environment hygiene, personal hygiene habits, exercise frequency, nap habits, etc.

#### 2.3.6 Dietary nutrition health status

Various self-designed items were included in the questionnaire to assess each subject’s eating habits, dietary attitudes and cognition, and recent dietary intake.

The reliability and validity of the above self-designed questions were investigated, with all reliability and validity indices above 80% (**Supplementary Table 3**).

### 2.4 Biological indicators

EDTA anticoagulation tubes were used to collect 5 mL venous blood samples from each participant. The blood samples were tested for HIV, syphilis and hepatitis C infection status.

#### 2.4.1 HIV antibody test

Initial screening of all samples was performed with Enzyme-Linked Immunosorbent Assay (ELISA-1). Positive samples were retested with another Enzyme-Linked Immunosorbent Assay (ELISA-2) from a different manufacturer or using different methodological principles to confirm the positive status.

#### 2.4.2 HIV antibody test

Zhuhai Lizhu reagent was used for the initial screening, and Beijing Wantai TRUST was used for the secondary screening.

#### 2.4.3 HCV antibody test

Samples were screened using ELISA-1. Those that returned a positive result on the initial screen were retested using ELISA-2 from a different manufacturer or using different methodological principles.

#### 2.4.4 Other biological indicators

Biological indicators related to sleep quality, anxiety, depression and other psychological problems were measured, including: corticotropin-releasing hormone (CRH), adrenocorticotropic hormone (ACTH), cortisol (COR), tumour necrosis factor-α (TNF-a), cyclooxygenase (COX-2), N terminal pro B type natriuretic peptide (NT-proBNP), fibroblast growth factor-21 (FGF-21), norepinephrine (NE/NA), γ-aminobutyric acid (GABA), melatonin (MT), α- synuclein (α-SYN), indolepropionic acid (IPA), etc.

### 2.5 Quality control

① Before the field investigation began, a small number of samples were selected for pre-investigation, in order to optimize the investigation process and improve the questionnaire. ② All staff involved in the investigation were trained in accordance with the requirements of the National AIDS Sentinel Monitoring Implementation Program. ③ The collected data were backed up locally every day, and 5% of the questionnaires from each survey site were selected for review. The scales that did not meet the research standards were excluded. ④ The double-entry method was adopted to avoid data entry errors. ⑤ Blood samples for research purposes were collected and stored in strict accordance with the relevant operating procedures and requirements.

### 2.6 Statistical methods

After double-entry checking using EpiData 3.1 statistical software, SAS 9.4 statistical software was utilised for data processing. Descriptive analysis was first performed. The numerical variables are presented by mean and standard deviation( x ± s); Categorical variable adoption rate or composition ratio and were compared by the χ2 test or Fisher’s exact text, the test level α was set at 0.05. Variables with P< 0.05 were included in the multi-factor logistic regression model, and the variables were screened by the forward stepwise method. The α levels of variables entering and eliminating were 0.05 and 0.1, respectively. A binary Logistic regression model was constructed to judge the predictive effects of each factor on sleep quality, anxiety and depression. Pearson correlation analysis was used to examine the correlations between depression, anxiety, sleep quality. In addition, In addition, multivariate regression analysis of sleep disorders was carried out to detect the influence of various biochemical indicators on sleep: an univariate logistic analysis was performed with sleep disorders as the dependent variable and the test levels of various biochemical indicators as the independent variables. The variables with significant differences in the univariate analysis were included in multi-factor logistic regression analysis to detect the risk factors associated with sleep disorders, and ROC curves were constructed, AUC was calculated, and the test level α was set at 0.1.

## 3 Results

### 3.1 Basic demographic characteristics of FSWs in Huanggang city

A total of 830 FSWs were surveyed, and 826 valid questionnaires were obtained (questionnaire response rate: 99.52%). The results indicated that most FSWs were married (70.1%) and not highly educated. Most lived in the county (82.9%), and their monthly income was most commonly 3000-5000 RMB. A small number of FSWs chose to live alone, while most FSWs chose to share a room (66.6%). In terms of lifestyle, more than half of the FSWs did not smoke cigarettes or drink alcohol; 78.6% of the FSWs did not engage in exercise (**Supplementary Table 4**).

### 3.2 Factors influencing depression among FSWs

The overall number of participants with depression was 270, with a prevalence rate of 32.7%. Among them, 143 (17.3%) were mildly depressed, 65 (7.9%) were moderately depressed, and 62 (7.5%) were severely depressed. The mean SDS standard score of FSWs was 47.74 ± 13.94 (**Table 1**).

**Table 1:** Distribution of symptoms of depression, anxiety and sleep quality.

Variable	scale	frequency	percentage(%)
Depression	non-depression(<53)	556	67.3
	mild depression(53-62)	143	17.3
	moderate depression(63-72)	65	7.9
	severe depression(>72)	62	7.5
Anxiety	non-anxiety(<50)	470	56.9
	mild anxiety(50-60)	344	41.6
	moderate anxiety(61-70)	8	1.0
	severe anxiety(>70)	4	0.5
Sleep Quality	normal sleep(≤7)	755	91.4
	somnipathy(>7)	71	8.6

The χ2 test or Fisher’s exact test was used to compare depression among FSWs with different marital statuses, residence locations, cohabitation numbers, education levels, income statuses, isolation, and living habits. The results showed that in terms of sociodemographic characteristics, the proportion of FSWs who were married, lived in the county, had a low level of education (junior high school or below), and had two or more cohabitants were higher in the non-depressed group than in the depressed group (P< 0.001), while FSWs whose monthly income exceeded 5000 RMB accounted for a higher proportion in the depressed group than in the non-depressed group. Regarding lifestyle habits, the proportion of FSWs who smoked, drank alcohol and exercise was higher in the depressed group than in the non-depressed group; in addition, the percentage of FSWs who had been isolated by the pandemic was higher in the depressed group (**Table 2**).

**Table 2:** Socio-demographics between depression group and non-depression group in FSWs.

Variable	Classification	Non-Depression (n=556) No.(%)	Depression (n=270) No.(%)	*χ2*	*P*
Marital Status	Spinsterhood	103(18.5)	91(33.7)	36.75	<0.001
	Married	427(76.8)	152(56.3)		
	Divorced or widowed	26(4.7)	27(10.0)		
Permanent Residence	Village or township	23(4.1)	25(9.3)	20.73	<0.001
	County	484(87.1)	201(74.4)		
	Urban	49(8.8)	44(16.3)		
Degree of Education	Primary School and below	82(14.7)	21(7.8)	29.25	<0.001
	Junior high school	311(55.9)	120(44.4)		
	Technical secondary school	70(12.6)	57(21.1)		
	Senior high school or above	93(16.7)	72(26.7)		
Income (yuan/month)	<3000	138(24.8)	60(22.2)	9.77	0.008
	3000-5000	240(43.2)	94(34.8)		
	>5000	178(32.0)	116(43.0)		
Number of Cohabitants	0	159(28.6)	117(43.3)	19.23	<0.001
	1	60(10.8)	30(11.1)		
	≥2	337(60.6)	123(45.6)		
Been Quarantined Due to the Pandemic	No	506(91.0)	205(75.9)	34.49	<0.001
	Yes	50(9.0)	65(24.1)		
Smoking Habit	No	387(69.6)	154(57.0)	12.70	<0.001
	Yes	169(30.4)	116(43.0)		
Drinking Habits	No	351(63.1)	116(43.0)	30.08	<0.001
	Yes	205(36.9)	154(57.0)		
Exercise Habit	No	455(81.8)	194(71.9)	10.76	<0.001
	Yes	101(18.2)	76(28.1)		

The factors that were statistically significant in the analyses, in addition to the SSRS score, were included as independent variables in a multi-factor logistic regression analysis; the coding of each factor is shown in **Supplementary Table 5**. The results showed that divorce or widowhood, secondary school education, alcohol consumption and exercise were independent risk factors for depression in FSWs (P< 0.05) while living in urban and county areas and having a sense of social support were protective factors for depression (P< 0.05) (**Table 3**).

**Table 3:** Multi-factor logistic regression analysis of depressed in FSWs in Huanggang City.

Variable	*β*	*S.E*	*Waldχ2*	*OR*	95%*CI*	*P*
Marital Status						
Married	0.40	0.25	2.52	1.50	0.91~2.44	0.112
Divorced/widowed	1.25	0.34	9.79	3.48	1.59~7.59	0.002
Permanent Residence						
county	-1.36	0.38	13.01	0.26	0.12~0.54	<0.001
urban	-1.08	0.45	5.70	0.34	0.14~0.82	0.017
Degree of Education						
Junior high school	0.26	0.33	0.63	1.30	0.68~2.46	0.429
Technical secondary school	0.91	0.38	5.77	2.48	1.18~5.20	0.016
Senior high school or above	0.61	0.37	2.70	1.85	0.89~3.83	0.1
**Drinking Habits**	0.70	0.20	11.74	2.00	1.35~2.98	0.001
**Exercise Habit**	0.50	0.22	5.33	1.65	1.08~2.54	0.021
**Social Support**	-0.22	0.02	165.97	0.81	0.78~0.83	<0.001

### 3.3 Factors influencing anxiety among FSWs

The total number of FSWs with anxiety was 356, with a prevalence rate of 43.1%. Among them, 344 (41.6%) had mild anxiety, 8 (1.0%) had moderate anxiety, and 4 (0.5%) had severe anxiety. The mean SAS standard score was 48.04 ± 8.21 (**Table 1**).

The χ2 test or Fisher’s exact test were used to compare the anxiety of FSWs with different marital statuses, residence locations, cohabitation numbers, education levels, income statuses, isolation, and living habits. The results showed that in terms of sociodemographic characteristics, the proportion of FSWs who were married, lived in the county, and had low education level (junior high school or below) was higher in the non-anxiety group than in the anxiety group (P< 0.001), and the proportion of FSWs whose monthly income exceeded 5000 RMB and living alone was higher in the anxiety group than in the non-anxiety group (P< 0.05); in terms of lifestyle habits, the proportion of FSWs who smoked, drank alcohol, and exercised daily was higher in anxiety group than in the non-anxiety group (P< 0.001); in addition, the proportion of FSWs quarantining due to the pandemic was higher in the anxiety group (P< 0.001) (**Table 4**).

**Table 4:** Socio-demographics between anxiety group and non-anxiety group in FSWs.

Variable	Classification	Non-Anxiety ,(n=470) No.(%)	Anxiety ,(n=356) No.(%)	*χ2*	*P*
Marital Status	Spinsterhood	70(14.9)	124(34.8)	10.76	0.001
	Married	374(79.6)	205(57.6)		
	Divorced or widowed	26(5.5)	27(7.6)		
Permanent Residence	Village or township	20(4.3)	28(7.9)	30.69	<0.001
	County	419(89.1)	266(74.7)		
	Urban	31(6.6)	62(17.4)		
Degree of Education	Primary School and below	69(14.7)	34(9.6)	21.93	<0.001
	Junior high school	266(56.6)	165(46.3)		
	Technical secondary school	58(12.3)	69(19.4)		
	Senior high school or above	77(16.4)	88(24.7)		
Income (yuan/month)	<3000	126(26.8)	72(20.2)	7.45	0.024
	3000-5000	193(41.1)	141(39.6)		
	>5000	151(32.1)	143(40.2)		
Number of Cohabitants	0	131(27.9)	145(40.7)	15.38	<0.001
	1	53(11.3)	37(10.4)		
	≥2	286(60.8)	174(48.9)		
Been Quarantined Due to the Pandemic	No	467(99.4)	244(68.5)	160.58	<0.001
	Yes	3(0.6)	112(31.5)		
Smoking Habit	No	345(73.4)	196(55.1)	30.18	<0.001
	Yes	125(26.6)	160(44.9)		
Drinking Habits	No	301(64.0)	166(46.6)	24.99	<0.001
	Yes	169(36.0)	190(53.4)		
Exercise Habit	No	390(83.0)	259(72.8)	12.58	<0.001
	Yes	80(17.0)	97(27.2)		

Factors that were statistically significant in the analyses above, in addition to the SSRS score, were included as independent variables in a multi-factor logistic regression analysis; the coding of each factor is shown in **Supplementary Table 5**. The results showed that quarantining due to the pandemic and exercise were independent risk factors for anxiety in FSWs (P< 0.05) while living in the county and having a sense of social support were protective factors (P< 0.05) (**Table 5**).

**Table 5:** Multi-factor logistic regression analysis of anxiety in FSWs in Huanggang City.

Variable	β	S.E	Wald*χ2*	OR	95%CI	*P*
**Permanent Residence**						
county	-1.20	0.42	8.12	0.3	0.13~0.69	0.004
urban	-0.60	0.52	1.32	0.6	0.20~1.52	0.25
**Been Quarantined Due to the Pandemic**	3.52	0.629	31.31	33.71	9.83~115.60	<0.001
**Exercise Habit**	0.61	0.25	6.17	1.84	1.14~2.98	0.013
**Social Support**	-0.31	0.03	142.61	0.73	0.70~0.77	<0.001

### 3.4 Factors influencing the sleep quality of FSWs

Sleep disorders were detected in 71 FSWs, with an overall prevalence rate of 8.6%. The mean PSQI score of FSWs was 3.54 ± 2.599 (**Table 1**).

The sleep quality in FSWs with different marital statuses, residence locations, cohabitation numbers, education levels, income statuses, isolation, and living habits was compared by χ2 test or Fisher exact test. The results showed that in terms of sociodemographic characteristics, the proportion of FSWs who lived in the county, had a low level of education (junior high school or below), and had two or more cohabitants was higher in the non-sleep disorder group than in the sleep disorder group (P< 0.05), and the proportion of unmarried FSWs was higher in the sleep disorder group than in the non-sleep disorder group (P< 0.001); in terms of lifestyle habits, the proportion of FSWs who smoked, drank alcohol, and exercised daily were higher in the sleep disorder group than in the non-sleep disorder group (P< 0.05); in addition, the proportion of FSWs quarantining due to the pandemic were higher in the anxiety group (P< 0.001). The difference in income status between the two groups was not statistically significant (**Table 6**).

**Table 6:** Socio-demographics between sleep disorders group and non-sleep disorders group in FSWs.

Variable	Classification	Non-sleep disorders, (n=755) No.(%)	sleep disorders , (n=71) No.(%)	χ2	*P*
Marital Status	Spinsterhood	156(20.7)	38(53.5)	39.00	<0.001
	Married	549(72.7)	30(42.3)		
	Divorced or widowed	50(6.6)	3(4.2)		
Permanent Residence	Village or township	39(5.2)	9(12.7)	7.73	0.021
	County	633(83.8)	52(73.2)		
	Urban	83(11.0)	10(14.1)		
Degree of Education	Primary School and below	95(12.6)	8(11.3)	17.05	0.001
	Junior high school	406(53.8)	25(35.2)		
	Technical secondary school	116(15.3)	11(15.5)		
	Senior high school or above	138(18.3)	27(38.0)		
Income (yuan/month)	<3000	178(23.6)	20(28.2)	0.81	0.666
	3000-5000	306(40.5)	28(39.4)		
	>5000	271(35.9)	23(32.4)		
Number of Cohabitants	0	240(31.8)	36(50.7)	17.10	<0.001
	1	78(10.3)	12(16.9)		
	≥2	437(57.9)	23(32.4)		
Been Quarantined Due to the Pandemic	No	690(91.4)	21(29.6)	206.71	<0.001
	Yes	65(8.6)	50(70.4)		
Smoking Habit	No	512(67.8)	29(40.8)	20.89	<0.001
	Yes	243(32.2)	42(59.2)		
Drinking Habits	No	441(58.4)	26(36.6)	12.54	<0.001
	Yes	314(41.6)	45(63.4)		
Exercise Habit	No	601(79.6)	48(67.6)	5.55	0.019
	Yes	154(20.4)	23(32.4)		

Factors that were statistically significant in the analyses above, in addition to the SSRS scale score, were included as independent variables in a multi-factor logistic regression analysis; the coding of each factor is shown in **Supplementary Table 5**. The results showed that quarantining due to the pandemic was an independent risk factor for sleep disorders in FSWs (P< 0.05) while being married was a protective factor (P< 0.05) (**Table 7**).

**Table 7:** Multi-factor logistic regression analysis of sleep quality in FSWs in Huanggang City.

Variable	β	S.E	Waldχ2	OR	95%CI	*P*
**Marital Status**						
married	-0.78	0.31	6.46	0.46	0.25~0.84	0.011
Divorced/widowed	-1.33	0.68	3.83	0.27	0.07~1.00	0.05
**Been Quarantined Due to the Pandemic**	3.05	0.30	102.29	21.20	11.73~38.32	<0.001

### 3.5 Correlation analysis between SDS, SAS, PSQI and SSRS of FSWs in Huanggang city

Pearson correlation analysis was performed between the SDS, SAS, PSQI and SSRS scores. The results showed that anxiety and depression were positively correlated, with a correlation coefficient of 0.72 (P< 0.01); PSQI scores were positively correlated with anxiety and depression, with correlation coefficients of 0.09 and 0.07, respectively (P< 0.05); social support was negatively correlated with anxiety, depression and PSQI scores, with correlation coefficients of -0.66, -0.58 and -0.09, respectively (**Table 8**).

**Table 8:** Pearson correlation analysis of SDS, SAS, PSQI and Social Support Scores.

scale	SDS	SAS	PSQI	Social support
**SDS**	1.00			
**SAS**	0.72**	1.00		
**PSQI**	0.07*	0.09*	1.00	
**Social support**	-0.58**	-0.66**	-0.09*	1.00

Note: *P<0.05,**P<0.01.

### 3.6 Relationships between sleep disorders and anxiety, depression and biological indicators

**Figure 1:**
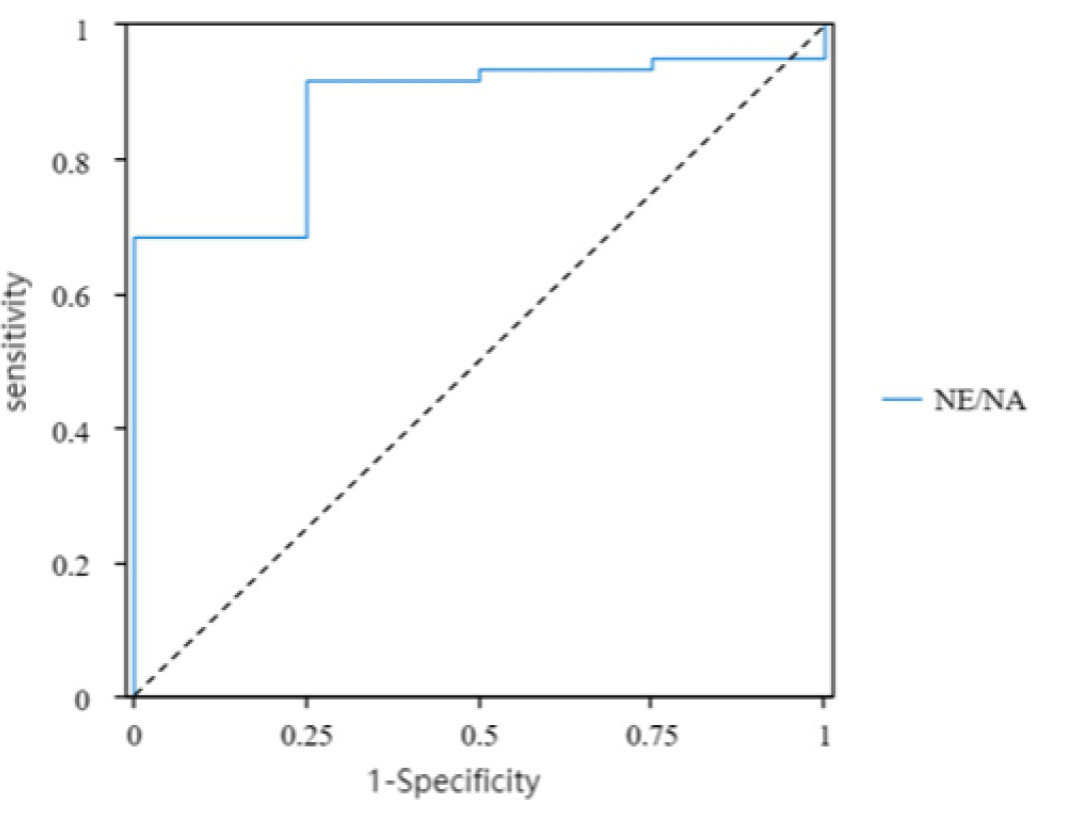
ROC of NE/NA and sleep disorders.

Ninety participants were randomly selected for fasting blood collection in order to detect relevant biological indicators. A total of 64 participants were included in the analyses, with an inclusion rate of 70.11%. Univariate logistic regression analysis was carried out with the presence of sleep disorders as the dependent variable and the various biological indicators as the independent variables. Variables that were significantly different in the univariate analyses were incorporated into a multivariate logistic regression model to detect the independent risk factors for sleep disorders. The α was set at 0.1. The results showed that, aside from NE/NA (P=0.05), there were no significant differences in the other biological indicators (CRH,ACTH,COR,TNF-a,COX-2,NT-proBNP,FGF-21,GABA,MT,α-SYN,IPA). The OR value for NE/NA was 0.04 (95%CI: 0.002-1.069) and the areas under the ROC curve (AUC) was 0.87 (**Figure 1**).

### 3.7 Knowledge and behavioural characteristics of FSWs in relation to hepatitis C and HIV prevention

Eight items in the questionnaire were about hepatitis C prevention and treatment and eight items were about AIDS-related knowledge. Those who answered six or more items correctly were defined as having good knowledge. The survey results indicated that 65 participants had good knowledge of hepatitis C prevention, with a rate of 7.87%. Further, 158 participants had good knowledge of AIDS prevention, with a rate of 19.13% (**Supplementary Tables 6, 7**).

In relation to the behavioural characteristics of FSWs, 95.5% reported using a condom every time they had sex with a client in the most recent month while 1.2% never used a condom in the most recent month. Further, 0.4% reported using drugs and 91.4% received services related to HIV prevention in the most recent year. Screening of FSWs for syphilis, HIV and hepatitis C was performed and all participants returned negative HIV antibody tests; 0.8% were positive for syphilis and HCV, but all were prior infections (**Supplementary Table 8**).

### 3.8 Knowledge of healthy diet and eating behaviours among FSWs

The questionnaire results revealed that 85.47% of FSWs knew the recommended daily intake of fruits was 200-350 g/d, 56.42% knew the recommended daily intake of oil was 25-30 ml, 92.74% knew the recommended daily intake of salt was<6 g, But the awareness rate of the recommended daily intake of water, potato and vegetables was about 30% (**Supplementary Table 9**). In relation to attitudes toward a healthy diet, 85% of FSWs thought that knowledge related to diet had a great impact on health and 75.4% valued their diet and health status; 84% thought that a healthy diet was more important in terms of healthy eating and meal preparation; 46.4% were willing to sacrifice some food taste for nutrition. Although most people focused on dietary nutrition, more than half of the participants (67.4%) did not actively learn about nutrition (**Supplementary Table 10**). The results in relation to the dietary behaviour of FSWs indicated that lunch was the meal in which most people (>80%) consumed the most staple foods, took the longest time, and ate the most nutritious meal; 91.04% of FSWs had a diet consisting of meat and vegetables; 71.19% used the “stir-fry” cooking method; 51.09% preferred bland foods, and 34.38% preferred spicy foods (**Supplementary Table 11**).

## 4 Discussion

FSWs engage in commercial sex service work; the occupation is unstable, and workers face various unique occupational situations, placing them at risk of psychological problems such as anxiety and depression. At the same time, because FSWs engage in more commercial sexual activities at night, they are more likely to experience sleep disorders. Insomnia is closely related to depression, anxiety and other mental disorders (21). If left untreated, mental health disorders can place women and their partners at risk of Human Immunodeficiency Virus/Sexually Transmitted Infections(HIV/STIs) (22).

In this study, 90 FSWs were randomly selected for biological index detection; seven of them (12.2%) had cortisol (COR) circadian rhythm disturbance. Moreover, corticotropin-releasing hormone (CRH), adrenocorticotropic hormone (ACTH) and COR concentrations were higher than normal. All these hormones are important molecules produced by the hypothalamus-pituitary-adrenal axis (HPAA) and are involved in the regulation of depressive disorders (23, 24). Changes in the COR circadian rhythm are an important sign of normal HPAA regulatory function (25). COR, as one of the most important end-products of HPAA, has a circadian cyclic pattern of secretion: it peaks at 6:00-8:00 am, then gradually declines, dropping to a minimum of less than 50% of the 8:00 am level at 0:00-2:00 pm. It then gradually rises again, reaching a peak within 1 h of waking, followed by an abrupt decline. After this, a slow downward trend continues throughout the day (26, 27). Normal COR levels are in a constant 24h cycle of change, following a circadian rhythm of variation. Cortisol, a major product of the HPAA axis, has multiple effects on the body. Dysregulated cortisol secretion can lead to many negative physical, cognitive and emotional problems (28–30). The circadian rhythms of specific hormones, such as COR, CRH, ACTH, melatonin and growth hormone-releasing hormone, play an important role in the distribution of these hormones throughout the sleep stages (30). The presence of abnormal rhythms in FSWs underlies the presence of insomnia and mood disorders from a molecular biological perspective. In addition, the results of this study showed that NE/NA was a protective factor for sleep disorders (OR=0.04, P=0.05), with an AUC of 0.87. This indicates it has a good diagnostic ability for sleep disorders.

In the current study, the rate of depression among FSWs was 32.7% and the rate of anxiety was 43.1%, which is higher than in the general population (12) and similar to the findings of Stockton et al. (31). A study in Iran reported that the prevalence of psychological problems among FSWs was 62.5%, with mood disorders and anxiety being the two most common problems; personality disorders were also observed in that study (5). The prevalence of psychological disorders among FSWs in Iran is slightly higher than that observed in the current study; this may be due to differences in the living environment and survival conditions of the two regions. Although FSWs are a low-income group in Huanggang, Wuhan, with the improvement of economic conditions and living standards, infrastructure construction and medical services are guaranteed, providing a good social environment for FSWs. The presence of a good social environment can reduce the incidence of psychological disorders (32).

In exploring the causes of psychological disorders in FSWs, it has been suggested that economic violence inflicted by customers on FSWs can lead to depressive symptoms. When negotiating prices in an informal way, customers tend to engage in economic violence and FSWs subsequently feel anxious or depressed when they believe they are not being paid enough for their services (7). On the other hand, most women choose to engage in commercial sex services because of poverty, which can increase their feelings of despair and insecurity. This, in turn, can lead to varying degrees of mental health disorders (6). A survey of FSWs in London showed that economically disadvantaged FSWs were more likely to experience anxiety or depression than the general population (33) (OR=3.66,95% CI=1.64 to 8.18). Moreover, a low income and an uncomfortable living environment reduced the quality of life of FSWs and affected their mental health status, while a good living environment improved the mental health status of FSWs. In the current study, multifactorial logistic regression analysis demonstrated that living in urban areas or counties was a protective factor for anxiety (OR=0.30, 95% CI=0.13-0.69) and depression (OR=0.34, 95% CI=0.14-0.82) among FSWs. Overall, 5.8% of the study participants lived in rural areas, 24% had a monthly income of less than 3000 RMB, and 66.6% chose to rent with others. Society should pay more attention to these factors in order to improve the quality of life of FSWs.

Infection with an STD increases the risk of developing psychological disorders. A trial in the Dominican Republic showed that FSWs with HIV/AIDS were more likely to be depressed, as compared to the general population of women without HIV/AIDS (4). Maclean concluded that mental health disorders can put FSWs and their partners at risk of HIV/STI infection (22). In the current study, all 826 study subjects tested negative for HIV; while 0.97% tested positive for syphilis and hepatitis C antigen, all were previously infected, indicating that the STD prevention and control efforts for FSWs in Huanggang City have been successful. Analysis of the knowledge and behaviours of the study subjects in relation to HIV and hepatitis C found that FSWs had generally poor knowledge of HIV and hepatitis C prevention (19.13% and 7.87%, respectively), and 1.2% had never used condoms in the last month. Condom use provides excellent protection for FSWs in terms of both health and economic security. In in-depth interviews with 35 FSWs in Bali, Indonesia, one study discovered that FSWs prioritize two factors, economic stability and romantic relationships, over condom use (34). A survey in South Africa showed that unprotected sex among alcoholics was 2.1 times more likely that among non-alcoholics (35). Malama et al. (36) concluded that pre-sex drinking behaviour increases the risk of HIV/AIDS infection, as FSWs who always consume alcohol prior to sex appear to be more likely to engage in riskier sexual behaviour. In the current study, 43.5% of FSWs consumed alcohol, and although the prevalence of HIV/AIDS infection among the participants was zero, multifactorial logistic regression showed that alcohol consumption was an independent risk factor for depressive symptoms, and drinkers were twice as likely as non-drinkers to be depressed (OR=2.00, 95% CI=1.35 to 2.98). Therefore, reducing alcohol use is an effective measure to improve the mental health status of FSWs and reduce the transmission of STDs.

Social isolation and health-related barriers are common for sex workers. In a study of Mexican migrant sex workers, peer support was noted to have an important role in preventing HIV/AIDS and violence (37). Peers are a valuable source of STD prevention knowledge and resources (e.g., condoms) for FSWs and an important safety support in the workplace. In this study, 91.4% of FSWs reported receiving peer education about HIV prevention and peer provision of condoms. Moreover, a sense of social support was a protective factor for anxiety (OR=0.73, 95% CI=0.70-0.77) and depression (OR=0.81,95% CI=0.78-0.83). However, the frequent mobility of sex workers makes it difficult for them to establish long-term stable social relationships, due to the unique nature of their occupation. Communities should pay more attention to the mobile FSW population by distributing free condoms to FSWs and increasing the frequency of STD prevention presentations in their workplaces. Marital status also affects the mental health status of FSWs and the risk of contracting STDs. A survey in one region of West Africa showed that single, divorced or widowed women were more likely to work in sex services (38), but in Huanggang, China, 70.1% of FSWs were married and only 29.9% were single, divorced or widowed. Thus, the situation in China is the exact opposite of the situation in West Africa. Moreover, divorce/widowhood was an independent risk factor for depressive symptoms in FSWs (OR=3.48,95% CI=1.59 to 7.59) and being married was a protective factor for sleep disturbances (OR=0.46,95% CI=0.25 to 0.84). An Indian study showed that widowed or divorced FSWs had a 2.73 times greater risk of HIV/AIDS infection than married FSWs (39), possibly because FSWs with spouses are concerned about transmitting STDs to their husbands and thus, are more protective of themselves at work.

In the present study, exercise was found to be an independent risk factor for anxiety (OR=1.84,95% CI=1.14 to 2.98) and depression (OR=1.65,95% CI=1.08 to 2.54) in FSWs. This is inconsistent with the results of previous related studies, where many studies have confirmed that exercise reduces the risk of mental health disorders in the general population (40–42). This finding may be related to specific occupational characteristics of FSWs. Specifically, this group may be more stringent than the general population in managing their body image and may be more likely to develop body image-related anxiety and depression if their body image does not meet their expectations. Additionally, more FSWs were aware of the recommended daily intakes of vegetables, fruits and oils, 75.4% attached importance to their diet and health status, and 84% considered healthy eating to be more important than entertaining, which indicates that FSWs attach importance to their physical health and will control their dietary intake to meet their physical requirements. FSWs faced new challenges in the context of the COVID-19 pandemic. FSWs were quarantined at home due to COVID-19, and their financial and physical health suffered (43). Multifactorial logistic regression analysis showed that FSWs quarantined due to the pandemic had a 33.71 times greater risk of anxiety (P< 0.001) and a 21.2 times greater risk of developing sleep disorders (P< 0.001) than those who were not quarantined. Data from an international sex work website reveals that, after a period of quarantine, sex workers are starting to work again, with a 9.4% increase in active sex workers (P<0.001) and a 35.6% increase in new sex workers (P<0.001) from May to August 2020 (44). An analysis of sex work and COVID-19 guidelines published in relevant regions indicates that most sex workers are focused on changing sexual behaviour, strengthening health and shifting to virtual work.

## 5 Conclusion

This study investigated the mental health status and sleep status of FSWs in Huanggang City and explored the relevant predictors. The results revealed that many FSWs experience poor mental health and the predictors of poor mental health are intertwined and complex. Marital status, economic status, HIV infection and sense of social support all affect the mental health and sleep quality of FSWs. It is noteworthy that the implementation of various policies in the context of the COVID-19 pandemic has greatly affected the work of FSWs, with the isolation and quarantine policy having the most pronounced impact. These results suggest that FSWs should appropriately adjust the form of their work to meet the new general environment, enhance their understanding of dietary nutrition, establish long-term stable social relationships, and seek a greater sense of social support. Moreover, the government should provide comprehensive bio-psycho-social interventions for this population, which, combined with the low percentage of condom use among this population for unsafe sex, could easily lead to the spread and spread of STD/AIDS if appropriate interventions are not taken in a timely manner. Warning education for this population should also be strengthened to raise their awareness of the risk of infection. In future work, targeted and easy-to-understand campaigns should be conducted for high-risk groups to effectively increase the knowledge rate of FSWs about HIV and hepatitis C, strengthen condom promotion, and reduce the spread of HIV and hepatitis C among FSWs.

## 6 Strengths and limitations

This study adopted a stratified cluster random sampling method to draw a whole-group random sample from different entertainment venues in Huanggang City in order to ensure that the results were representative. Moreover, the questionnaire addressed the mental health status and sleep quality of FSWs in Huanggang City, and STD infection-related data were also collected. The findings revealed correlations between depression, anxiety, sleep quality and social support among FSWs. Further, serological tests were performed to determine the biological indicators that are associated with sleep quality. Finally, this comprehensive study attempted to identify a wide range of factors affecting the mental health status of FSWs, including lifestyle behaviours, physical health status and social support.

However, there are some limitations of this research that should be noted. First, the study was a cross-sectional survey, and thus, can only present information on the current mental health status of FSWs and the possible influencing factors; causal relationships between mental health and the various influencing factors cannot be assumed. Second, in the detection of biological indicators, due to the small sample size, false negative results may occur. As a result, it is impossible to determine the effect of non-statistically significant biological indicators on sleep quality. Moreover, the high mobility and low adherence of FSWs, in addition to the hidden nature of the profession, make it difficult to implement effective research on comprehensive psychological interventions. Although the government has increased its investment in HIV prevention and control measures in recent years, with high priority given to sexual transmission, existing research and interventions have mostly focused on the control of high-risk behaviours. Little research has been conducted on the emotional issues affecting high-risk behaviours, and there is a lack of studies on psychological interventions to reduce high-risk sexual behaviours among FSWs.

## Data availability statement

The original contributions presented in the study are included in the article/**Supplementary Material**. Further inquiries can be directed to the corresponding authors.

## Ethics statement

Written informed consent was obtained from the individual(s) for the publication of any potentially identifiable images or data included in this article.

## Author contributions

JC and JZ conceived and designed the study. PZ, HL and YZ drafted the paper and finalized the manuscript. TH, CX, YL, GW, XC, JT, CJ performed literature review and proofreading. All authors contributed to discussions and the final draft. All authors contributed to the article and approved the submitted version.
