# Supplementary material for: Correlation analysis between sleep quality and the mental health status of female sex workers during the COVID-19 pandemic in Hubei Province
Source: Front Endocrinol (Lausanne). 2023 Jul 17;14:1193266. doi: 10.3389/fendo.2023.1193266 (PMC10388539; doi:10.3389/fendo.2023.1193266)
Supplement: Supplementary file 1 [file Table_1.docx]

# Supplementary tables

| C01.Whether a person infected with hepatitis C virus may not have any abnormal feelings and symptoms, must be tested to detect? | ① Yes ② no ③ unknown |
| --- | --- |
| C02.Does having sex with multiple individuals increase the risk of hepatitis C infection? | ① Yes ② no ③ unknown |
| C03.Does adherence to proper condom use reduce the risk of infection and transmission of hepatitis C? | ① Yes ② no ③ unknown |
| C04.Can sharing a syringe infect hepatitis C? | ① Yes ② no ③ unknown |
| C05.Can the blood with hepatitis C virus infect hepatitis C? | ① Yes ② no ③ unknown |
| C06.Can tattoos, eyebrows and ear piercing possibly infect hepatitis C? | ① Yes ② no ③ unknown |
| C07.Can chronic hepatitis C possibly develop for liver cirrhosis, liver cancer? | ① Yes ② no ③ unknown |
| C08.Can the liver C be cured? | ① Yes ② no ③ unknown |

**Supplementary table 1 Knowledge on rate of hepatitis C prevention and treatment in FSWs**

**Supplementary table 2 AIDS prevention knowledge awareness questionnaire of FSWs**

| C01 Is AIDS an incurable and serious infectious disease? | ① Yes ② no ③ unknown |
| --- | --- |
| C02 Is sexual transmission the main mode of transmission of AIDS in China? | ① Yes ② no ③ unknown |
| C03 Can we judge whether a person is infected with AIDS by the genital appearance? | ① Yes ② no ③ unknown |
| C04 Does contracting other STDs increase the risk of contracting AIDS? | ① Yes ② no ③ unknown |
| C05 Does adherence to proper condom use reduce the risk of infection and AIDS transmission? | ① Yes ② no ③ unknown |
| C06 Does the use of new drugs (such as methamphetamine, ecstasy, K powder, etc.) increase the risk of AIDS infection? | ① Yes ② no ③ unknown |
| C07 Should you actively seek AIDS testing and counseling after high-risk behavior (drug sharing drugs / unsafe sex, etc.)? | ① Yes ② no ③ unknown |
| C08 Does the intentional transmission of AIDS bear legal responsibility? | ① Yes ② no ③ unknown |

**Supplementary table 3 Adult health status questionnaire**

| **PartⅠ, the basic situation** | | | | | | | | | |
| --- | --- | --- | --- | --- | --- | --- | --- | --- | --- |
| A1 | | name： | | | | | | | |
| A2 | | contact information | | ①phone code ②QQ ③Wechat | | | | | |
| A3 | | ethnic groups | | ① Han ② Tujia ③ Zhuang ④ Miao ⑤ Hui ⑥ Tibetan ⑦ other ethnic groups | | | | | |
| A4 | | date of birth | | □□□□,□□,□□ | | | | | |
| A5 | | native place | | ① Hubei ② Hunan ③ Henan ④ Guizhou ⑤ Yunnan ⑥ Sichuan ⑦ Guangxi ⑧ Anhui ⑨ Shaanxi ⑩ Chongqing Other provinces | | | | | |
| A6 | | Marital status | | ① Unmarried ② cohabitation ③ married ④ divorced ⑤ widowed | | | | | |
| A7 | | Permanent location | | ① Village ② Township ③ county ④ city | | | | | |
| A8 | | degree of education | | ① Illiterate ② primary school ③ junior high school ④ ⑤ high school ⑥ college ⑦ bachelor degree or above | | | | | |
| A9 | | Household income (the farmer's annual income is divided by 12) | | ① Less than 1000 yuan a month ② 1000~3000 yuan a month ③ 3000~5000 yuan a month ④ 5000~7000 yuan a month ⑤ 7000~9000 yuan a month ⑥ 9000 yuan a month or above | | | | | |
| A10 | | How many children do you have？ | | ① 0 ② 1 ③ 2 ④ 3 ⑤ 4 ⑥ 5 and above | | | | | |
| A11 | | How many people live with you? | | ① 0 ② 1 ③ 2 ④ 3 ⑤ 4 ⑥ 5 and above | | | | | |
| A12 | | Who are the members that you live with?(Can be selected more) | | ① Grandparents (including grandparents) ② parents (including parents-in-law) ③ spouse ④ brothers and sisters ⑤ children (including daughter-in-law and son-in-law) ⑥ grandchildren (including grandchildren) ⑦ other | | | | | |
| A13 | | What are your daily interests?(Can be selected more) | | ① None ② chess and cards ③ shopping ④ sleeping ⑤ doing housework or farm work ⑥ dancing ⑦ fitness ⑧ ball games ⑨ gymnastics ⑩ others | | | | | |
| A14 | | Have you ever tested positive for COVID-19 or an asymptomatic infected? | | ① Yes, once positive ② yes, once asymptomatic infected person ③ no | | | | | |
| A15 | | Have you ever been quarantined because of the outbreak? | | ①Yes ②no | | | | | |
| A16 | | Have you ever suffered from depression? | | ①Have ever suffered from ②In the disease ③Never suffer from | | | | | |
| A17 | | Whether you take the related medication? | | ①No ②Yes (Write down the relevant drugs and doses) | | | | | |
|  | |  | |  | | | | | |
| **Part II: The Behavior and Lifestyle Survey** | | | | | | | | | |
| **Quality of life** | | | | | | | | | |
| B1 | Do you have the habit of smoking? | | | | | | | | ① No, turn to B4 ② has had and has quit, turn to B4 ③ is quitting smoking ④ smoking addiction ⑤ smoking e-cigarettes |
| B2 | If you smoke, smoke a few a day | | | | | | | | ① 1~2 ② 3~5 ③ 6~10 ④ with 11 branches or more |
| B3 | How long did you smoke | | | | | | | | 1. <1 year ② 1~3 years ③ 3~10 ④ more than 10 years |
| B4 | Are you exposed to people with current smokers?？ | | | | | | | | ① Yes, turn to B5 ② no, turn to B6 |
| B5 | How much is the number of days that you are exposed to second-hand smoke per week?(Second-hand smoke is smoke exhaled by a smoker and emitted from the end of a cigarette) | | | | | | | | ① Daily ② average 4-6 days per week ③ average 1-3 days per week ④ no ⑤ unknown / do not remember |
| B6 | What is the health condition of your living environment? | | | | | | | | ① Very good ② better ③ very bad ④ worse |
| B7 | Do you wash your hands carefully before meals and after using the toilet every day? | | | | | | | | ① Every time ② often ③ occasionally ④ never |
| B8 | Do you live in a place with a strong noise? | | | | | | | | ① Yes, turn to B8 ② no, turn to B9 |
| B9 | How long the noise lasts | | | | | | | | ①Under 8 hours ② 8-9 hours ③ 9-10 hours ④ More than 10 hours |
| B10 | Do you have the habit of drinking alcohol?(For the next three answers, all turn to B11) | | | | | | | | ① No, turn B13 ② once had and has quit ③ yes and is quitting drinking ④ yes |
| B11 | What are the types of alcohol you drink?(Can be selected more) | | | | | | | | ① Liquor ② red wine ③ beer ④ fruit wine ⑤ rice wine |
| B12 | How much do you drink per day / week? | | | | | | | | ① A small cup, approximately 50-100 ml ② 100~250 ml ③ 250 ml ~500 ml ④ 500 ml or more |
| B13 | Do you have the habit of drinking tea?(For the next three answers, all turn to B14) | | | | | | | | ① No, turn B16 ② once had, has quit ③yes, is quitting ④yes |
| B14 | What is the type of tea you drink?(Can be selected more) | | | | | | | | ① White tea ② black tea ③ green tea ④ dark tea ⑤ yellow tea ⑥ oolong tea |
| B15 | How much tea do you drink every day / week？ | | | | | | | | ① 1 cup (about 500ml) ② 2~5 cups ③ 5 ~10 cups ④ 10 cups above |
| B16 | Do you have a habit of drinking coffee?(For the next three answers, all turn to B18) | | | | | | | | ① No, turn B16 ② once had, has quit ③yes, is quitting ④yes |
| B17 | What kind of coffee do you drink?(Can be selected more) | | | | | | | | ① Espresso ② mocha coffee ③ Americano ④ latte ⑤ cappuccino ⑥ Miso |
| B18 | How much coffee do you drink per day / per week? | | | | | | | | ① 1 cup (about 500ml) ② 2~5 cups ③ 5 ~10 cups ④ 10 cups above |
|  |  | | | | | | | |  |
| **Physical activity** | | | | | | | | | |
| B19 | What do you think of your usual health condition? | | | | | | | ① Very good, energetic ② is ok, occasionally feel tired ③ is poor, low spirit | |
| B20 | Do you do any daily exercise and exercise?(Including running, swimming, gymnastics, etc., excluding daily activities such as walking and walking) | | | | | | | ① Almost no (if choose this item, jump to B22) ② January 1-3 times ③ 1-3 times a week ④ almost every day | |
| B21 | What is the main form of your usual exercise? | | | | | | | ① Running ② swimming ③ ball games ④ rope skipping ⑤ outdoor sports with friends ⑥ dancing ⑦ yoga/pilates ⑧ fitness ⑨ aerobics ⑩ others | |
| B22 | Which attitude do you take most of you in your daily life and work? | | | | | | | ① Most of the time sitting ② most of the time lying ③ most of the time standing ④ most of the time in motion | |
| B23 | What are the main entertainment activities for you after work? | | | | | | | ① Indoor activities (computer, mobile phone games, watching TV, chess and cards, etc.) ② Outdoor activities (travel, picnic, ball ball, shopping, etc.) | |
|  |  | | | | | | |  | |
| **Sleep quality** | | | | | | | | | |
| B24 | Do you think you have enough sleep? | | | | | | ① Very sufficient ② Quite sufficient ③ is not enough ④ is far from enough | | |
| B25 | Do you have the habit of staying up late? | | | | | | ① Stay up late almost every day ② Occasionally stay up late ③ never stay up late | | |
| B26 | Is there a habit of napping? | | | | | | ① Yes, to B30 ② no , to C1 | | |
| B27 | How long does each nap last? | | | | | | ① 30 minutes ② 1 hour ③ 2 hours ④ 2 hours or more | | |
| B28 | Have you doze off during the night in the past month？ | | | | | | ① Very few (0-5 days) ② few (6~12 days) ③ sometimes (13~18 days) ④ often (19~24 days) ⑤ always (25~31 days) | | |
| B29 | How is your mood after insomnia? | | | | | | ① No discomfort ② no matter ③ sometimes upset, impatient ④ palpitation, shortness of breath ⑤ fatigue, no spirit, low efficiency | | |
|  |  | | | | | |  | | |
| **Part Ⅲ, the diet situation** | | | | | | | | | |
| **Eating habits** | | | | | | | | | |
| C1 | How many times do you usually eat a day? | | | | | ① 1 ② 2 ③ 3 ④ 4 ⑤ 5 or more | | | |
| C2 | Is the meal time regular? | | | | | ① Daily meal is basically the same time ② occasionally not according to the usual meal time ③ no specific meal time | | | |
| C3 | Can morning, lunch, lunch and dinner eat on time? | | | | | ① Yes ② occasionally can not ③ occasionally can ④ completely can not | | | |
| C4 | Who cooks the food?(Except for the school canteen) | | | | | ① Parents ② Grandparents ③ Takeaway ④ Other________ | | | |
| C5 | Do you usually eat at where? | | | | | ① Home ② canteen ③ Road ④ restaurant ⑤ classroom ⑥ transport on ⑦ Other | | | |
| C6 | How many people do you usually eat with you? | | | | | ① Only one person ② two people ③ three people ④ four or more | | | |
| C7 | Do you have the habit of eating midnight snacks? | | | | | ① Always ② often ③ occasionally ④ never | | | |
| C8 | Do you eat takeout? | | | | | ① Always ② often ③ occasionally ④ never | | | |
| C9 | Do you have the habit of snacking outside of dinner time? | | | | | ①Yes, turn to C10 ②No, turn to C11 | | | |
| C10 | Do you think eating snacks is more meaningful than eating meals? | | | | | ①Yes ②No | | | |
| C11 | Do you eat the fruit after the dinner? | | | | | ① Always ② often ③ occasionally ④ never | | | |
| C12 | Which flavor of food is more attractive to you?(multiple choice) | | | | | ① Sweet ②spicy ③ sour ④ salty ⑤ bitter ⑥ light | | | |
| C13 | What kind of cooking food is more attractive to you?(multiple choice) | | | | | ① Stir-fried ② fried ③ steamed ④ brine vegetables ⑤ barbecue ⑥ smoked vegetables ⑦ Stew dish ⑧ salad | | | |
| C14 | What do you like at dinner? | | | | | ① Vegetables ② meat and egg milk ③ vegetables and meat and egg milk | | | |
| C15 | Do you eat fruit all day? | | | | | ① Always ② often ③ occasionally ④ never | | | |
| C16 | How much water do you drink in a day? | | | | | ① Almost once do not drink ② drink 1-4 cups ③ drink 5-8 cups ④ drink more than 8 cups | | | |
| C15 | Do you drink milk tea? | | | | | ① Always, every day will eat and drink ② often, 3-5 days a week ③ occasionally, 1-2 days a week ④ never, to C18 | | | |
| C16 | How sweet is the milk tea you often drink? | | | | | ① Free ② tragon ③ pentagon ④ sevenagon ⑤ whole sugar | | | |
| C17 | Do you think that drinking milk tea has an impact on your health? | | | | | ①not at all ②Sometimes think so ③  Fully agree | | | |
| C18 | Do you eat dessert every day (lollipop, chocolate, cake) every day? | | | | | ① Always, every day will eat ② often, 3-5 days a week ③ occasionally, 1-2 days a week ④ never | | | |
| C19 | Are you eating a latiao snack every day? | | | | | ① Always, every day will eat ② often, 3-5 days a week ③ occasionally, 1-2 days a week ④ never | | | |
| C20 | Do you have a weight loss plan? | | | | | ① Yes, and in the weight loss program ② no, but not the weight loss program ③ no | | | |
| C21 | Do you feel too hungry to eat? | | | | | ① Always (almost every meal do not eat) ② often ③ occasionally (occasionally a meal to eat) ④ never | | | |
| C22 | Do you think snacks can replace meals? | | | | | ①Yes ②no | | | |
|  |  | | | | |  | | | |
| **Dietary attitude and cognitive situation** | | | | | | | | | |
| **Nutrition function and food sources** | | | | | | | | | |
| C23 | The major food source of vitamin D | | | | ① Grain grain ② vegetables ③ fruits ④ poultry meat ⑤ fish and shrimp ⑥ eggs, milk | | | | |
| C24 | The main food source of nitrogen | | | | ① Grain grain ② vegetables ③ fruits ④ poultry meat ⑤ fish and shrimp ⑥ eggs, milk | | | | |
| C25 | The main food source of calcium | | | | ① Grain grain ② vegetables ③ fruits ④ poultry meat ⑤ fish and shrimp ⑥ eggs, milk | | | | |
| C26 | The effect of the lack of vitamin A on the body | | | | ① Fatigue ② numbness of limbs and legs ③ sore body ④ night blindness, edema and shortness of breath | | | | |
| C27 | The lack of vitamin B2 on the body | | | | ① Seborrheic dermatitis ② stomatitis ③ anemia ④ diarrhea ⑤ glossitis ⑥ rickets | | | | |
| C28 | The effect of excessive sodium intake on the body | | | | ① Muscle weakness ② increased blood pressure ③ muscle spasm ④ burnout ⑤ vomiting ⑥ blood pressure drop | | | | |
| **Food value** | | | | | | | | | |
| C29 | What role do the nutrients play | | ① Maintenance of physiological activity ② hyperplasia new tissue ③ production energy ④ involved in body metabolism ⑤ unclear | | | | | | |
| C30 | Nutrients that the human body needs | | ① Carbohydrates ② fat ③ protein ④ vitamin ⑤ water inorganic salts | | | | | | |
| C31 | Fruit provides nutrients | | ① Carbohydrates ② fat ③ protein ④ vitamin water inorganic salts | | | | | | |
| C32 | Which vitamin has a therapeutic effect on beriberi | | ① Vitamin A ② Vitamin B1 ③ vitamin B2 ④ vitamin D ⑤ vitamin C ⑥ unknown | | | | | | |
| C33 | Which foods can cause high uric acid | | ① Beer ② seafood ③ mushrooms ④ bean products ⑤ poultry meat ⑥ unknown | | | | | | |
| C34 | Which substance can prevent iron deficiency | | ① Pig, beef, mutton ② liver, blood ③ milk powder ④ eggs ⑤ fruits, vegetables ⑥ unknown | | | | | | |
| **Dietary guidelines** | | | | | | | | | |
| C35 | Recommended daily water intake | | | ①500-1,200mL ②1,200-1,500mL ③1,500-1,700mL ④1,700-2,000mL ⑤Above 2,000 mL | | | | | |
| C36 | Recommended daily intake of cereal potatoes | | | ① 0-100 g ② 100-150 g ③ 250-400 g ④ 400-550 g ⑤ 550 g or more | | | | | |
| C37 | Recommended daily intake of vegetables | | | ① 0-100 g ② 100-300 g ③ 300-500 g ④ 500-700 g ⑤ 700 g or more | | | | | |
| C38 | Recommended daily intake of fruit | | | ① Under 100 g ② 100-200 g ③ 200-350 g ④ 350-500 g ⑤ 500 g or more | | | | | |
| C39 | Recommended daily intake of cooking oil | | | ① 0-10 g ② 10-25 g ③ 25-30 g ④ 30-45 g ⑤ 45 g or more | | | | | |
| C40 | Recommended daily salt intake | | | ① <3 g ② <6 g ③ <9 g <12 g ⑤ <15 g | | | | | |
|  |  | | |  | | | | | |
| **Healthy meal attitude** | | | | | | | | | |
| C41 | Do you think diet-related knowledge has health implications? | | | | | ① Large impact ② small effect ③ has no effect | | | |
| C42 | Do you value your diet and health status? | | | | | ①Value them ②don't value them ③don't pay attention to this problem | | | |
| C43 | What do you choose between healthy eating and social dinner? | | | | | ① Healthy diet is more important ②for the career can not follow a reasonable healthy diet ③ both unimportant | | | |
| C44 | What is your choice for taste and nutritional value? | | | | | ① Low appetite nutritional value does not matter ② high nutritional value can sacrifice some taste ③ taste and nutritional value I want | | | |
| C45 | Will you take the initiative to learn more about nutrition | | | | | ①Yes ②no ③don't pay attention to this problem | | | |
|  |  | | | | |  | | | |
| **Dietary habits** | | | | | | | | | |
| C46 | Do you have breakfast every day? | | | | | ① Never eat breakfast, turn C49 ② for breakfast, turn C47 | | | |
| C47 | When do you probably have your breakfast? | | | | | ①5:00-6：00 ②6:00-7：00 ③7:00-8：00 ④8:00-9：00 | | | |
| C48 | What is the staple food for your general breakfast? | | | | | ① Meal replacement food ② noodles ③ steamed bread ④ bread ⑤ rice ⑥ others | | | |
| C49 | When do you probably have your lunch | | | | | ①11:00-12：00 ②12:00-13：00 ③13:00-14：00 ④ after 14:00 | | | |
| C50 | What is the main staple food for your lunch? | | | | | ① Meal replacement food ② noodles ③ steamed bread ④ bread ⑤ rice ⑥ others | | | |
| C51 | When do you probably have your dinner? | | | | | ①18:00-19：00 ②19:00-20：00 ③20:00-21：00 ④ after 21:00 | | | |
| C52 | What is your staple food for dinner? | | | | | ① Meal replacement food ② noodles ③ steamed bread ④ bread ⑤ rice ⑥ others | | | |
| C53 | Which meal do you eat the staple food the most three times a day? | | | | | ① Breakfast ② lunch ③ dinner | | | |
| C54 | Which one of your three meals a day does you take the longest time? | | | | | ① Breakfast ② lunch ③ dinner | | | |
| C55 | Which of you eat the most nutrients at three meals a day (excluding the staple food)? | | | | | ① Breakfast ② lunch ③ dinner | | | |
| C56 | What is your diet structure? | | | | | ① With meat based ② vegetable based ③ meat and vegetable collocation | | | |
| C57 | Which cooking method do you prefer? | | | | | ① Steam ② stir-fry ③ stew ④ fried ⑤ others ( ) | | | |
| C58 | What kind of food do you like to eat？ | | | | | ① Sweet ② salty ③ spicy ④ acid ⑤ light | | | |
| C59 | Do you have the habit of eating a midnight snack snack？ | | | | | ① Often ② occasionally ③ never, turn C61 | | | |
| C60 | If you have a midnight snack, usually when? | | | | | ①21:00-22：00 ②22:00-23：00 ③Other time periods | | | |
| C61 | Which meals do you eat at home every day (optional)? | | | | | ① Breakfast ② lunch ③ dinner ④never eat at home | | | |
| C62 | How many days do you usually store your leftovers for? | | | | | ① No leftovers ②work it out in one day ③ one day ④ two days ⑤ three days or more | | | |
| C63 | Do you think your diet is regular? | | | | | ① Regular ② irregular | | | |

**Supplementary table 4 Basic demographic characteristics of the FSWs**

| **variable** | **classify** | **frequency** | **Percentage(%)** |
| --- | --- | --- | --- |

| Marital status | Unmarried | 194 | 23.5 |
| --- | --- | --- | --- |
|  | Married | 579 | 70.1 |
|  | Divorce or Widowed | 53 | 6.4 |
| Permanent location | Villages or towns | 48 | 5.8 |
|  | county seat | 685 | 82.9 |
|  | urban area | 93 | 11.3 |
| degree of education | Primary school and below | 103 | 12.5 |
|  | junior middle school | 431 | 52.2 |
|  | polytechnic school | 127 | 15.4 |
|  | High school and above | 165 | 20 |
| Income (yuan / month) | Less than 3000 | 198 | 24 |
|  | 3000-5000 | 334 | 40.4 |
|  | 5000 and above | 294 | 35.6 |
| Number of people living together | 0 | 276 | 33.4 |
|  | 1 | 90 | 10.9 |
|  | 2 and above | 460 | 55.7 |
| Have you ever been quarantined because of the outbreak? | Yes | 115 | 13.9 |
|  | No | 711 | 86.1 |
| Is there a habit of smoking? | No | 541 | 65.5 |
|  | Yes | 285 | 34.5 |
| Is there a habit of drinking alcohol? | No | 467 | 56.5 |
|  | Yes | 359 | 43.5 |
| Do you do any daily exercise or exercise? | No | 649 | 78.6 |
|  | Yes | 177 | 21.4 |

**Supplementary table 5 Variables and assignments**

| **variable** | **assignment** |
| --- | --- |
| Melancholy | 1=Yes；0=No |
| Anxiety | 1=Yes；0=No |
| Dyssomnia | 1=Yes；0=No |
| Marital status | 0=Unmarried；1=Married；2=Divorce or Widowed |
| Permanent location | 0=Villages and towns；1=County seat；2=City |
| Degree of education | 0=Primary school and below；1=Junior middle school；2=Polytechnic school ；3=High school and above |
| Drink | 1=Yes；0=No |
| Daily exercise | 1=Yes；0=No |
| Social support | 1=Yes；0=No |
| Isolation due to the epidemic | 1=Yes；0=No |

**Supplementary table 6 Knowledge of hepatitis C prevention and treatment in FSWs（n=826）**

| **Knowledge of hepatitis C control in FSW population** | **Number of people known** | **awareness rate（%）** |
| --- | --- | --- |
| Whether a person infected with hepatitis C virus may not have any abnormal feelings and symptoms, must be tested to detect? | 89 | 10.77 |
| Does having sex with multiple individuals increase the risk of hepatitis C infection? | 83 | 10.05 |
| Does adherence to proper condom use reduce the risk of infection and transmission of hepatitis C? | 79 | 9.56 |
| Can sharing a syringe infect hepatitis C? | 71 | 8.60 |
| Can the blood with hepatitis C virus infect hepatitis C? | 75 | 9.08 |
| Can tattoos, eyebrows and ear piercing possibly infect hepatitis C? | 65 | 7.87 |
| Can chronic hepatitis C possibly develop for liver cirrhosis, liver cancer? | 67 | 8.11 |

**Supplementary table 7 Awareness of AIDS knowledge among FSWs（n=826）**

| **AIDS knowledge** | **Number of people known** | **awareness rate（%）** |
| --- | --- | --- |
| Is AIDS an incurable and serious infectious disease? | 332 | 40.19 |
| Is sexual transmission the main mode of transmission of AIDS in China? | 192 | 23.24 |
| Can we judge whether a person is infected with AIDS by the genital appearance? | 40 | 4.84 |
| Does contracting other STDs increase the risk of contracting AIDS? | 177 | 21.43 |
| Does adherence to proper condom use reduce the risk of infection and AIDS transmission? | 182 | 22.03 |
| Does the use of new drugs (such as methamphetamine, ecstasy, K powder, etc.) increase the risk of AIDS infection? | 147 | 17.80 |
| Should you actively seek AIDS testing and counseling after high-risk behavior (drug sharing drugs / unsafe sex, etc.)? | 175 | 21.19 |
| Does the intentional transmission of AIDS bear legal responsibility? | 175 | 21.19 |

**Supplementary table 8 Behavioral characteristics and intervention profile of the FSWs**

| **Behavior-related entries** | **Example number** | **percentage (%)** |
| --- | --- | --- |
| **Did you use a condom when you last had sex with a guest?** |  |  |
| Yes | 800 | 96.9 |
| No | 26 | 3.1 |
| **How often do you use condoms when having sex with your guests in the last month?** |  |  |
| Never used | 10 | 1.2 |
| Sometimes use | 27 | 3.3 |
| Use it every time | 789 | 95.5 |
| **Do you take drugs?(Including heroin, cocaine, opium, marijuana, morphine, methamphetamine, demerol, K powder / chloride, ecstasy, ephedrine)** |  |  |
| Yes | 3 | 0.4 |
| No | 823 | 99.6 |
| **Have you received the following AIDS prevention services in the past year?** |  |  |
| **1. Condom publicity and distribution / AIDS counseling and testing** |  |  |
| Yes | 755 | 91.4 |
| No | 71 | 8.6 |
| **2. Companion education** |  |  |
| Yes | 755 | 91.4 |
| No | 71 | 8.6 |
| **Syphilis test results: ELISA** |  |  |
| positive | 7 | 0.8 |
| negative | 819 | 99.25 |
| **RPR/TRUST test** |  |  |
| positive | 7 | 0.8 |
| negative | 819 | 99.25 |

**Supplementary table 9 Knowledge of the dietary guidelines**

| **class** | **Recommended Intakes (g/d or ml)** | **Number of people known** | **awareness rate（%）** |
| --- | --- | --- | --- |
| Water | 1500-1700 | 264 | 31.96 |
| Valley potato class | 250-400 | 267 | 32.32 |
| Vegetables | 300-500 | 267 | 32.32 |
| Fruit | 200-350 | 706 | 85.47 |
| Edible oil | 25-30 | 466 | 56.42 |
| Salt | <6 | 766 | 92.74 |

**Supplementary table 10 Attitude related to a healthy diet**

| **Dietary attitude-related entries** | **frequency** | **percentage (%)** |
| --- | --- | --- |
| **Do you think diet-related knowledge has health implications?** |  |  |
| Large impact | 702 | 85 |
| Small effect | 70 | 8.5 |
| Has no effect | 54 | 6.5 |
| **Do you value your diet and health status?** |  |  |
| Value them | 623 | 75.4 |
| Don't value them | 157 | 19 |
| Don't pay attention to this problem | 46 | 5.6 |
| **What do you choose between healthy eating and social dinner?** |  |  |
| Healthy diet is more important | 694 | 84 |
| For the career can not follow a reasonable healthy diet | 95 | 11.5 |
| Both unimportant | 37 | 4.5 |
| **What is your choice for taste and nutritional value?** |  |  |
| Low appetite nutritional value does not matter | 99 | 12 |
| High nutritional value can sacrifice some taste | 383 | 46.4 |
| Taste and nutritional value I want | 344 | 41.6 |
| **Will you take the initiative to learn more about nutrition?** |  |  |
| Yes | 222 | 26.9 |
| No | 557 | 67.4 |
| don't care | 47 | 5.7 |

**Supplementary table 11 Dietary habits**

| **Dietary habits entry** | **Frequency** | **percentage (%)** |
| --- | --- | --- |
| **Which meal do you eat the staple food the most three times a day?** |  |  |
| Breakfast | 1 | 0.12 |
| Lunch | 722 | 87.41 |
| Dinner | 103 | 12.47 |
| **Which one of your three meals a day does you take the longest time?** |  |  |
| Breakfast |  |  |
| Lunch | 719 | 87.05 |
| Dinner | 107 | 12.95 |
| **Which of you eat the most nutrients at three meals a day (excluding the staple food)?** |  |  |
| Breakfast | -- | -- |
| Lunch | 766 | 92.74 |
| Dinner | 60 | 7.26 |
| **What is your diet structure?** |  |  |
| With meat based | 10 | 1.21 |
| Vegetable based | 64 | 7.75 |
| Meat and vegetable collocation | 752 | 91.04 |
| **Which cooking method do you prefer?** |  |  |
| Steam | 29 | 3.51 |
| Stir-fry | 588 | 71.19 |
| Stew | 1 | 0.12 |
| Fried | 208 | 25.18 |
| **What kind of food do you like to eat？** |  |  |
| Sweet | 59 | 7.14 |
| Salty | 39 | 4.72 |
| Spicy | 284 | 34.38 |
| Acid | 22 | 2.66 |
| Light | 422 | 51.09 |
